# Assessing the Structural Validity of the Knee Injury and Osteoarthritis Outcome Score Scale

**DOI:** 10.3390/healthcare12040414

**Published:** 2024-02-06

**Authors:** Dylan T. Quintana, Madeline P. Casanova, Adam C. Cady, Russell T. Baker

**Affiliations:** 1 WWAMI Medical Education Program, University of Idaho, Moscow, ID 83844, USA; dquintan@uw.edu (D.T.Q.); mcasanova@uidaho.edu (M.P.C.); 2 Idaho Office of Underserved and Rural Medical Research, University of Idaho, Moscow, ID 83844, USA; 3 Kaiser Permanente, Woodland Hills, CA 91367, USA; adam.c.cady@kp.org

**Keywords:** knee pathology, psychometric analysis, factor analysis, patient-reported outcome measure

## Abstract

Background: The Knee Injury and Osteoarthritis Outcome Score (KOOS) scale is used to assess patient perspectives on knee health. However, the structural validity of the KOOS has not been sufficiently tested; therefore, our objective was to assess the KOOS in a large, multi-site database of patient responses who were receiving care for knee pathology. Methods: A cross-sectional study was conducted using the Surgical Outcome System (SOS) database. A confirmatory factor analysis (CFA) was conducted to assess the proposed five-factor KOOS using a priori cut-off values. Because model fit indices were not met, a subsequent exploratory factor analysis (EFA) was conducted to identify a parsimonious model. The resulting four-factor structure (i.e., KOOS SF-12) was then assessed using CFA and subjected to multigroup invariance testing. Results: The original KOOS model did not meet rigorous CFA fit recommendations. The KOOS SF-12 did meet model fit recommendations and passed all invariance testing between intervention procedure, sex, and age groups. Conclusion: The KOOS failed to meet model fit recommendations. The KOOS SF-12 met model fit recommendations, maintained a multi-factorial structure, and was invariant across all tested groups. The KOOS did not demonstrate sound structural validity. A refined KOOS SF-12 model that met recommended model fit indices and invariance testing criteria was identified. Our findings provide initial support for a multidimensional KOOS structure (i.e., KOOS SF-12) that is a more psychometrically sound instrument for measuring patient-reported knee health.

## 1. Introduction

Clinicians use various measures to make informed decisions on patient care, assess patient progress, examine treatment effectiveness, identify areas for improvement, and improve healthcare outcomes [1,2]. Data reflecting the patient’s perspective of their health status, collected via patient-reported outcome measures (PROMs), is critical in treatment evaluation. Clinicians gain valuable insight using PROMs, and these data are important for assessing treatment effectiveness or informing patient-centered care [2,3,4]. PROMs may also be multidimensional (e.g., generic, body region-specific, injury-specific) to gain a more holistic view of the patient’s health status [4,5].

One commonly used multidimensional PROM is the Knee Injury and Osteoarthritis Outcome Score (KOOS). The KOOS is a 42-item self-administered questionnaire designed to assess short- and long-term patient-relevant outcomes for knee pain and osteoarthritis [3]. The KOOS was designed by a panel of experts (e.g., physical therapists, patients, orthopedic surgeons) reviewing items from the Western Ontario and McMaster Universities Osteoarthritis Index (WOMAC). Items were revised, and a pilot study was conducted using 21 patients (9 males and 12 females) undergoing ACL reconstruction to provide a measure of patient knee symptoms and function after inciting trauma and to follow their subsequent recovery [6]. The KOOS has since been broadly used in research and clinical settings [3] across various patient populations (e.g., pediatric, geriatric, ACL tear, meniscus tear) and languages (e.g., Arabic, Chinese, Swedish) [6,7,8,9].

Despite its broad use, only preliminary evidence of the psychometric properties of the KOOS has been established [10,11,12,13]. For example, researchers have examined convergent and divergent construct validity by correlating KOOS scores to the SF-36 and the Lysholm knee scoring scale [6]. Additionally, pooled values for Cronbach’s α have also been reported, with values ranging between 0.70 and 0.95 for all subscales, and reliability has been tested using intra-class correlation coefficients (ICCs), with values ranging from 0.85 to 0.9 (I^2^ ≥ 68.9%) [10]. Lastly, moderate construct validity was reported based on principal component analysis [7,14].

Many of these measurement properties require further testing; others (e.g., measurement invariance) have not yet been established, and concerns have been raised about the KOOS [10]. For example, construct validity of subscales has been called into question [13]: partial Rasch analysis indicated only two subscales of the KOOS met unidimensional measurement scale requirements [11], and the reported Cronbach’s alpha levels (i.e., values ≥ 0.90) indicated item redundancy and internal consistency concerns that require further scale modification [15,16]. Additionally, studies assessing the structural validity of the KOOS have often failed to follow best-practice recommendations for samples (e.g., small sample sizes) or data analysis (e.g., using principal components analysis instead of exploratory factor analysis [EFA] and confirmatory factor analysis) [10,17,18,19,20]. Thus, sound conclusions regarding the proposed five-factor (i.e., latent variable) structure of the KOOS cannot be made [10].

Additional research is necessary to establish the structural validity of the KOOS. Specifically, examining the factor structure of the scale using CFA procedures and CFA-based invariance testing is necessary to establish structural validity and justify scale use in clinical practice and research [17,19,21,22]. Therefore, the purpose of this study was to utilize a large, multi-site database of patient responses to assess the structural validity of the KOOS using CFA procedures. Because model fit criteria were not met, the secondary purpose of this study was to identify a justifiable solution that retained the theoretical multi-dimensional KOOS model using EFA-based procedures. The identified model was then assessed using CFA-based (i.e., covariance modeling) procedures and then assessed for invariance between subgroups of interest (i.e., intervention procedure, sex, and age groups).

## 2. Materials and Methods

### 2.1. Data Source and Participants

De-identified patient responses to the KOOS were obtained from the Surgical Outcome System (SOS), a patient-reported outcome database maintained by Arthrex (Naples, FL, USA). The SOS adheres to Health Insurance Portability and Accountability Act (HIPAA) regulations, and patients provide informed consent prior to using the SOS. The University Institutional Review Board (IRB) reviewed the project and indicated IRB approval was not required; however, IRB approval was provided by the Cedar-Sinai Office of Research Compliance and Quality Improvement. English-speaking patients who completed the KOOS after presenting to a provider with complaints of knee pain/dysfunction were included and were separated by the care received (i.e., arthroplasty, arthroscopy, or non-operative [i.e., non-op]). A breakdown of intervention groups from the SOS database is presented in Table 1.

### 2.2. KOOS Instrumentation

The KOOS is a 42-item PROM composed of 5 subscales: symptoms (7 items; Ex: “Do you have swelling in your knee”), pain (9 items; Ex: “Straightening knee fully”), activities of daily living (17 items; Ex: “What difficulty have you experienced the last week rising from sitting?”), sports and recreation (5 items; Ex: “What difficulty have you experienced the last week jumping?”), and knee-related quality of life (4 items; Ex: “How often are you aware of your knee problems?”) [3]. Each item is rated using a Likert scale from 0 (no problems) to 4 (extreme problems) [3].

### 2.3. Data Analysis

#### 2.3.1. Data Cleaning

Data were analyzed using Statistical Package for the Social Sciences (SPSS, Inc., Chicago, IL, USA) Version 26.0 and Analysis of Moment Structure (AMOS, SPSS, Inc., Chicago, IL, USA) Version 25.0. All individuals with missing responses to KOOS items were deleted; however, individuals with missing demographic data were retained. Data cleaning included the assessment of univariate distributions of variables to verify a normal distribution with low levels of skewness/kurtosis. Assessment of multivariate outliers was conducted using Mahalanobis distance with a cut-off value identified in the chi-square table with degrees of freedom and a p value of 0.01. The full sample was split into two random samples (n1 = 2000; n2 = 2001). Sample n1 was used to conduct a CFA of the five-factor KOOS. Sample n1 was also used to conduct EFAs to identify a parsimonious solution meeting model fit criteria. Sample n2 was used to conduct a covariance model on the EFA solutions identified. The full sample was then used to conduct multi-group invariance testing between surgical, sex, and age groups on the identified solution.

#### 2.3.2. Confirmatory Factor Analysis

Sample n1 was used to conduct a CFA using maximum likelihood estimation in AMOS. Model fit indices were based on a priori values to evaluate factor structures. The relative goodness-of-fit indices computed included the Comparative Fit Index (CFI; >0.95), Tucker–Lewis Index (TLI; >0.95), Root Mean Square Error of Approximation (RMSEA < 0.06), and Bollen’s Incremental Fit Index (IFI; >0.95). The likelihood ratio statistic (Chi square, or CMIN) was also assessed but not used as the primary assessment to model fit. Localized areas of strain in the solution were assessed, and the interpretability, size, and statistical significance of the model’s parameter estimates (i.e., factor variances, covariances, and indicator errors) were reviewed [21].

#### 2.3.3. Exploratory Factor Analysis

The five-factor KOOS did not meet the model fit criteria. Using sample n1, EFAs with maximum likelihood extraction and direct oblimin rotation were conducted. Bartlett’s test of sphericity (<0.001) and Kaiser–Meyer–Olkin values (≥0.80) were assessed [16,18]. Item evaluation to guide item removal consisted of using survey design principles to assess item content, theory, and structure [18,23], as well as item loadings (≥0.40), item cross-loadings (≤0.30), inter-item correlations (≥0.80), and low internal consistency [16,18]. Four criteria were used to determine factor retention: (1) Eigenvalue ≥ 1.0; (2) scree plot inflection point examination; (3) factors accounted for ≥5.0% of the variance in the data; and (4) parallel analysis results [16,24]. Cronbach’s alpha and omega (ω) were set a priori at ≥0.70 and ≤0.89 [16,25,26].

#### 2.3.4. Covariance Model on Proposed Models

The proposed KOOS models identified during the EFA process were then assessed using covariance modeling (i.e., CFA-based procedures) in sample n2. The same model fit criteria used for the initial CFAs were used to assess these models. Scores on the original KOOS and the proposed subscales were correlated to scores on the newly proposed final model and the corresponding subscales of the short form; the cut-off for an acceptable amount of variance between the scales was set a priori at r ≥ 0.90 and R^2^ = 0.81 [27].

#### 2.3.5. Invariance Testing on the Final Proposed Model

The full sample was used to conduct multigroup invariance testing across surgical procedure groups, sexes, and age groups using AMOS. The same model fit criteria used for the CFA procedures were assessed, and structural, metric, and scalar invariance models were tested [19]. The CFI difference test (>0.01; CFIDIFF) and the chi-square difference test (χ^2^_DIFF_; using a *p*-value cut-off of 0.01) were used to determine invariance between groups [21,28]. Both criteria were assessed; however, the CFIDIFF test held greater weight in decisions regarding model fit [19,21], and testing would continue if the χ^2^_DIFF_ was exceeded but the CFIDIFF was not.

## 3. Results

A sample of 5858 individuals was randomly selected from the SOS database. A total of 1252 individuals were missing one or more responses from the KOOS and were deleted from the dataset. Additionally, a total of 183 univariate outliers and 422 multivariate outliers were identified and removed from the dataset. A total of 4001 individuals were included in the final analysis. The participant’s age ranged from 12 to 89 years (mean age = 54.41 ± 16.49 years), with females accounting for 53% (n = 2132) and males accounting for 45% (n = 1787) of the sample. Participant demographics by intervention group are presented in Table 2.

### 3.1. Confirmatory Factor Analyses

**Figure 1:**
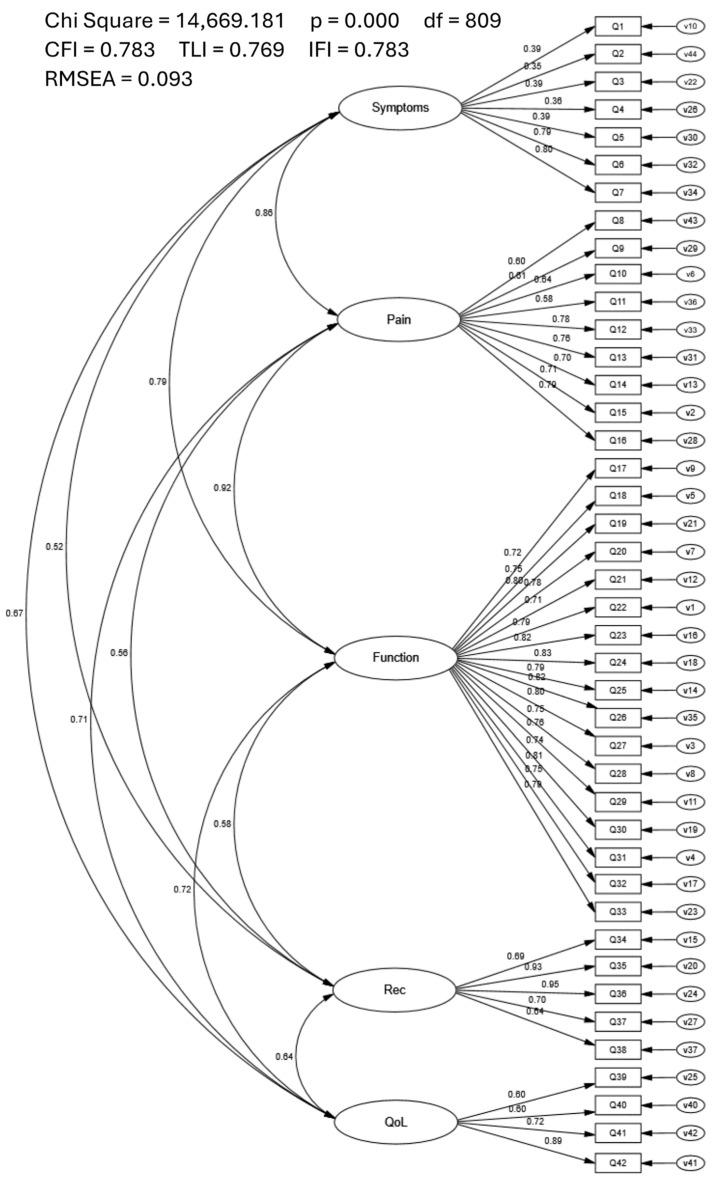
Confirmatory Factor Analysis of the Five-Factor KOOS.

The CFA goodness-of-fit indices of the five-factor KOOS did not meet the recommended values (CFI = 0.783; TLI = 0.769; IFI = 0.783; RMSEA = 0.093; Figure 1). Although all factor loadings were significant (range = 0.35–0.95), correlations between latent variables (e.g., Pain, Function) were high, ranging from r = 0.52 to r = 0.92. Furthermore, modification indices suggested numerous meaningful cross-loadings were present.

### 3.2. Exploratory Factor Analysis: Identification of a 4-Factor Model

The EFA of the KOOS using sample n1 initially extracted six factors with eigenvalues > 1.0. The solution included items with low loadings and high cross-loadings, and parallel analysis only supported five factors. Item removal followed the described methodology until a 4-factor, 12-item model (KOOS SF-12; 75.67% of the variance explained) was identified (Table 1). Parallel analysis supported a two-factor structure with those 12 items; however, the four-factor solution met the other three a priori criteria and was therefore retained. Factor 1 consisted of three items from the original ‘Pain’ factor and retained the name ‘Pain.’ Factor two consisted of three items from the original ‘Recreation’ factor and retained the name ‘Recreation.’ Factor three consisted of three items from the original ‘Function’ factor and retained the name ‘Function.’ Factor four consisted of three items from the original ‘Quality of Life’ (QOL) factor and retained the name ‘QOL.’ Cronbach’s alpha and omega values were within the acceptable range (Table 3).

### 3.3. Confirmatory Factor Analysis: Covariance Modeling of the 4-Factor Model

**Figure 2:**
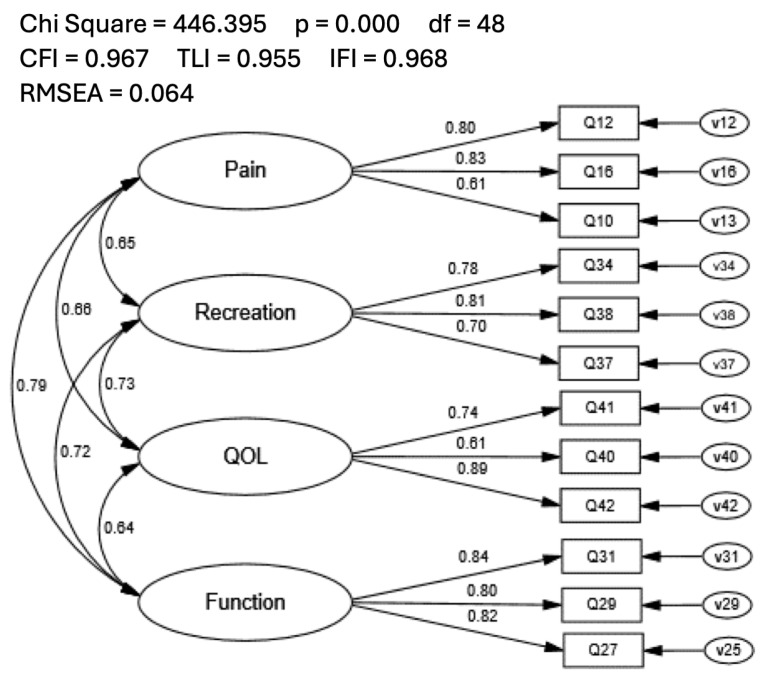
Confirmatory Factor Analysis of the 4-factor KOOS Short Form 12-item Solution.

The proposed 4-factor solution (i.e., KOOS SF-12) was then assessed in a covariance model using CFA procedures with sample n2. The fit indices for the KOOS SF-12 met all model fit recommendations (CFI = 0.967; TLI = 0.955; IFI = 0.968; RMSEA = 0.064; Figure 2). Individual item loadings ranged from 0.60 to 0.90, and inter-factor correlations were high, ranging from 0.61 to 0.89. Modification indices suggested numerous meaningful cross-loadings were present, which included all items in the QOL factor. Participant scores for the original KOOS were highly correlated (r = 0.959, R^2^ = 0.920) with participant scores from the KOOS SF-12. Additionally, subscale scores from the KOOS were highly corrected (Pain: r = 0.912, R^2^ = 0.832; Function: r = 0.915, R^2^ = 0.837; Recreation: r = 0.948, R^2^ = 0.899; QOL: r = 0.986, R^2^ = 0.972) with participant subscale scores of the KOOS SF-12. The high correlation values indicated that participant responses on the KOOS SF-12 explained an acceptable amount of variance in responses on the original KOOS and the associated subscales.

### 3.4. Multi-Group Invariance Testing of the Proposed KOOS SF-12

#### 3.4.1. Intervention Procedure Groups

The full sample (n = 40,001) was used for the intervention procedure group analysis (arthroplasty = 1341, arthroscopy = 1445, non-operative = 1215). Individual models for treatment groups met model fit indices (Table 4). The configural model met model fit indices (CFI = 0.967, RMSEA = 0.036). The metric model passed invariance criteria, warranting analysis of an equal variance model. The equal variance model passed invariance criteria, indicating variances were not statistically different between groups. The scalar model did not pass invariance criteria, thus not allowing analysis of an equal means model. Further inspection of the model indicated item #37 in the ‘Recreation’ factor (i.e., “Twisting/pivoting on your injured knee”) and item #42 in the ‘QOL’ factor (i.e., “In general, how much difficulty do you have with your knee”) were exhibiting slight bias. When both items were not restricted, invariance criteria were met.

#### 3.4.2. Sex Groups

A total of 3919 individuals reported sex (male = 1787, female = 2132) and were used for analysis. Individual models for males and females met model fit indices (Table 5). The configural model met model fit indices (CFI = 0.966, RMSEA = 0.047). The metric model and scalar models passed invariance criteria, warranting analysis of equal variance and equal means model. The equal variance model passed invariance criteria, indicating variances were not statistically different between groups. The equal means model also passed invariance testing, indicating the means were not statistically different between sexes.

#### 3.4.3. Age Groups

A total of 3218 individuals reported their age (middle-aged adults [41–65 years] n = 2145; older adults [66+ years] n = 1073) and were used for analysis. Individual models for middle-aged adults and older adults met model fit indices (Table 6). The configural model met model fit indices (CFI = 0.970, RMSEA = 0.063). Both the metric model and scalar model passed invariance criteria, warranting analysis of an equal variance and equal means model. The equal variance model passed invariance criteria, indicating variances were not statistically different between groups. The equal means model also passed invariance testing, indicating the means were not statistically different between age groups.

### 3.5. Confirmatory Factor Analysis: KOOS-12

**Figure 3:**
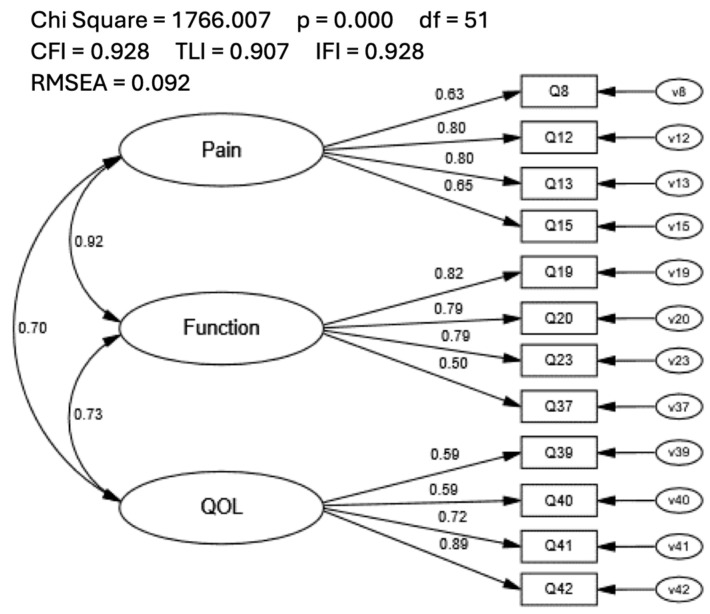
Confirmatory Factor Analysis of the 3-Factor, 12-item KOOS.

Because the analysis yielded a 12-item model, we opted to compare the model fit of the KOOS SF-12 in the current study to a previously proposed 3-factor, 12-item short-form KOOS (i.e., KOOS-12 [29,30]) to provide insight into scale selection between the two scales. The KOOS-12 was assessed in a covariance model using CFA procedures with the full sample. Fit indices for the KOOS-12 did not meet model fit recommendations (CFI = 0.928; TLI = 0.907; IFI = 0.928; RMSEA = 0.092; Figure 3). Individual item loadings ranged from 0.50 to 0.89, and inter-factor correlations were high, ranging from 0.70 to 0.92. Modification indices suggested numerous meaningful cross-loadings were present, which included all items in the QOL factor.

## 4. Discussion

The purpose of this study was to explore the structural validity and invariance properties of the KOOS using a large, multi-site database to guide its use in clinical practice and in research. CFA results revealed the KOOS had poor model fit, significant correlations between latent variables, and numerous meaningful cross-loadings. Thus, the secondary purpose was to identify a justifiable multi-dimensional KOOS structure via EFA. A 12-item, 4-factor model (i.e., KOOS SF-12) was found and subjected to covariance modeling and multi-group invariance testing. The proposed KOOS SF-12 performed significantly better, meeting model fit recommendations. and multigroup invariance criteria across intervention procedures, sexes, and age groups.

Several weaknesses of the KOOS were identified. For example, the KOOS contains double-barreled items (i.e., asking two [or more] questions with a single item), nonmonotonic items (i.e., items that could be answered for two or more different reasons), and similar items that overlap across proposed dimensions. Surveys designed with these items have greater ambiguity and confusion for respondents [23,31], as well as increased response burden and reduced measurement precision [32]. The survey structure and item design likely account for much of the poor model fit, while item ambiguity, item overlap, and reduced measurement precision can be identified in the high correlations between latent variables (e.g., pain, symptoms, function). The higher correlations found between these constructs are evidence of multicollinearity bordering on singularity, suggesting patients are unable to differentiate between the items intended to measure different constructs. Thus, a similar phenomenon is likely being measured across the KOOS items/latent variables as opposed to the intended unique latent variables (e.g., pain, symptoms) [19,21].

Prior research [14], which reported inflated Cronbach’s alpha levels across the pain, function (i.e., activities of daily living), and recreation/sports constructs, combined with other findings [10], has been suggestive of item redundancy and construct overlap. Our correlational findings, as well as the numerous cross-loadings found during our CFA, further support these concerns. These findings, along with poor model fit, suggest the originally proposed KOOS does not have sound structural validity; thus, multi-group and longitudinal invariance testing on the original KOOS was not warranted. Therefore, either rewriting, adding, or removing items from the KOOS may produce an item set more likely to measure the intended factors and improve model fit [19,32]. We chose to perform EFA with item removal to examine if a parsimonious and sound model could be identified within the available data because prior research [7,10,14] had indicated potential for other multidimensional KOOS solutions.

Our exploratory approach was informed by best practice recommendations for item reduction in EFA and follow-up CFA-based procedures [17,18,19,21]; however, we retained as many of the original factors as possible to reflect the intended design and purpose of the KOOS [6] and because measuring multiple constructs within a single instrument is beneficial [4,33]. The EFA process identified a 4-factor, 12-item model (i.e., KOOS SF-12) that reflected the initial multi-dimensional KOOS model but with improved internal consistency and scale structure (e.g., reduced multicollinearity, reduced response burden, improved model fit, etc.). Development of the KOOS SF-12 primarily occurred through the removal of 30 items with low loadings, high cross-loadings, high correlation values, poor item design (e.g., double-barreled items), or a combination of these factors.

The KOOS SF-12 measures four of the five proposed constructs (i.e., pain, quality of life, function, and sport/recreation) and reduces patient response burden while increasing measurement precision. The CFA-based procedures applied to the KOOS SF-12 demonstrated sound model fit that met strict fit criteria recommendations. Additionally, although the KOOS SF-12 only retained 29% of the items from the original KOOS, total scores and subscale scores between the two instruments were highly correlated. Thus, participant scores on the KOOS SF-12 accounted for a substantial amount of the variance in the responses to the original KOOS and indicate a similar phenomenon is being measured despite the removal of more than 70% of the items [27]. While the results indicate sound structural validity, it should be noted that measurement concerns (e.g., cross-loadings) and survey design concerns are still present in the KOOS SF-12. These issues are also present in the previously proposed KOOS-12; however, the newly proposed KOOS SF-12 had reduced measurement concerns and more sound psychometric properties when compared to the previously proposed KOOS-12 [29,30].

The EFA and CFA results for the KOOS SF-12, unlike the CFA results of the KOOS or KOOS-12, were strong enough to support multi-group invariance testing to determine if items and constructs were interpreted and operationalized in the same manner across sex, age, and intervention procedure groups (i.e., arthroplasty, arthroscopy, and non-operative). Multigroup invariance testing provides additional insight for clinicians and researchers as it supports scale use for hypothesis testing and group difference assessment [19,21,28]. Multigroup analysis for intervention procedure groups passed the metric model requirement but not the scalar model requirements, which allowed for group variance comparison with the KOOS SF-12; statistical differences for group variances were not found. The KOOS SF-12 metric model, however, did not pass invariance testing requirements; thus, it would be inappropriate to compare treatment group mean differences on the KOOS SF-12. We identified two biased items that would result in measurement errors across the groups. Caution should be used when comparing mean score differences between treatment groups on the KOOS SF-12, as it cannot be guaranteed that those group differences are true differences and did not result from error (e.g., how an item is interpreted) [19,21,28].

In contrast, sex multi-group invariance testing passed all criteria. Therefore, clinicians and researchers can compare group differences across means and variances for males and females with the KOOS SF-12. The only other multigroup invariance testing of the KOOS or its alternate versions identified in the literature occurred with the KOOS-JR. Prior researchers [34] indicated the KOOS-JR was invariant across sexes, and statistically significant mean differences between males and females were found at baseline examination. Statistically significant differences were not found at the initial examination in the current study, which may be due to differences in samples or the KOOS SF-12 containing more dimensions or items than the KOOS-JR (7 items for 1 dimension). Normative KOOS reference data has indicated adult males and females report statistically different scores for the pain and symptoms constructs [33]; however, it is unknown how the inclusion of biased or problematic items may have introduced measurement error into those findings. As sex differences (e.g., females having a lower capacity to cope with musculoskeletal pain [35], males having higher kinesiophobia or lower activity levels for similar pain levels [36]) have been reported, future research using the KOOS SF-12 is warranted to determine when those group differences arise and what may account for those differences.

Multi-group invariance testing was also performed across different age groups, and the KOOS SF-12 was found to be invariant. The KOOS SF-12 met invariance criteria, which indicates the KOOS SF-12 can be used to assess perceived knee health across the age groups in this study (i.e., middle-aged [41–65 years], older adults [66+ years]), and differences found reflect true group differences as opposed to measurement error. Statistically significant latent mean or variance differences, however, were not found. Our results contrast prior studies, which found that older age groups reported greater knee health impairments on the KOOS [37,38] and that perceived knee impairment increased across the lifespan [37,38,39]. Notably, our results are exclusively based on a sample of injured patients, as opposed to prior studies that used healthy patients or attempted to create population-based reference data. Sample differences or scale differences may explain the contrasting results. Future work should be performed to clarify if statistically significant differences in perceived knee health across the life span are found with the KOOS SF-12.

### Limitations and Future Research

While the present study has many strengths (e.g., a large sample size), limitations exist. First, all psychometric (e.g., reliability, longitudinal invariance testing, etc.) and subgroup analyses relevant to clinical practice and research were not able to be performed. Specifically, data were not available to conduct multigroup invariance testing by age, patient activity level, and other surgical procedures, which would offer valuable insight to clinicians treating diverse groups of patients for knee pathology. Therefore, caution is warranted when examining KOOS SF-12 score differences in groups not analyzed. Future research should assess the invariance properties of more relevant subgroups as well as their properties across time.

Further, the available data did not have KOOS responses from healthy individuals (preventing comparison to controls), detailed demographic information (preventing multigroup invariance testing by additional subgroups), or longitudinal data (preventing longitudinal psychometric analysis). Understanding whether patients interpret items of the KOOS and the newly developed KOOS SF-12 consistently across time would allow providers to confidently track perceived knee health throughout the treatment process. Future research should assess the longitudinal invariance properties of the KOOS SF-12 and confirm the criterion validity of the proposed subdimensions of the KOOS SF-12. Additionally, all relevant measurement properties of the KOOS SF-12 were not tested in our study, and future research on scale reliability (e.g., minimal detectable change) and responsiveness (e.g., minimal clinically important differences) would be valuable. Finally, the creation of new items or the alteration of existing items or Likert response options may allow for further reduction of measurement imprecision and better measurement of the intended subscales; future research should explore these options to create a more psychometrically sound multi-dimensional instrument to measure knee health.

## 5. Conclusions

The original KOOS model did not meet rigorous CFA model fit recommendations. Subsequent EFA led to the identification of the KOOS SF-12, which met the most recommended model fit indices and invariance testing criteria. Our findings offer initial support for the use of the KOOS SF-12 as a more psychometrically sound instrument than the KOOS or KOOS-12. The KOOS SF-12 resembles the initial KOOS structure and adequately measures the same phenomenon; thus, it can be used in clinical and research settings for similar purposes as the original KOOS. However, survey design concerns remain, and future research is needed to conduct further subgroup analyses and longitudinal invariance testing with the new KOOS SF-12 to guide the proper use of the scale in clinical practice and research.

## Figures and Tables

**Table 1:** SOS database group breakdown. Number of individuals screened for inclusion criteria and included in the initial sample, broken down by intervention group.

	Intervention Group		
	Arthroplasty n (%)	Arthroscopy n (%)	Non-Operative n (%)
Total n = 5858	2000 (34.14%)	2000 (34.14%)	1858 (31.72%)

**Table 2:** Participant demographics. Demographic characteristics of participants by intervention group.

Intervention Group n (% of Total Sample)	Age (Years) M ± SD	Males n (%)	Females n (%)	Sex Unknown n (%)
Arthroplasty 1341 (34%)	64.55 ± 8.85	594 (44.30%)	744 (55.48%)	3 (0.22%)
Arthroscopy 1445 (36%)	40.32 ± 16.20	680 (47.06%)	710 (49.13%)	55 (3.81%)
Non-operative 1215 (30%)	60.04 ± 10.70	513 (42.22%)	678 (55.80%)	24 (1.98%)

**Table 3:** Exploratory factor analysis. Exploratory factor analysis of the KOOS SF-12.

Item	Function	QOL	Pain	Recreation
Q27	0.847			
Q31	0.790			
Q29	0.764			
Q41		0.865		
Q40		0.626		
Q42		0.515		
Q38			0.845	
Q34			0.735	
Q37			0.649	
Q12				0.894
Q16				0.790
Q10				0.353
**Eigenvalue (% variance)**	5.92 (49.32)	1.33 (11.07)	0.96 (7.96)	0.75 (6.28)
**Cronbach’s Alpha**	0.86	0.79	0.81	0.80
**Omega**	0.86	0.80	0.81	0.79

**Table 4:** Measurement invariance for intervention procedure groups. Measurement invariance of the KOOS SF-12 across intervention procedure groups.

	χ^2^	df	χ^2^_DIFF_ (df_DIFF_)	CFI	CFI_DIFF_	TLI	IFI	RMSEA
Arthroplasty (n = 1341)	261.345	48	--	0.966	--	0.953	0.966	0.058
Arthroscopy (n = 1445)	331.701	48	--	0.970	--	0.959	0.970	0.064
Non-operative (n = 1215)	312.514	48	--	0.966	--	0.953	0.966	0.067
Configural Model	905.563	144	--	0.967	--	0.955	0.968	0.036
Metric Model	976.042	160	70.479 (16)	0.965	0.002	0.957	0.965	0.036
Equal Variances	1088.710	168	183.147 (24)	0.961	0.006	0.954	0.961	0.037
Scalar Model	1491.054	176	585.491 (32)	0.944	0.023	0.937	0.944	0.043
Equal Latent Means	NT	NT	NT	NT	NT	NT	NT	NT

χ^2^_DIFF_ *p* < 0.01 or CFI_DIFF_ > 0.01; NT = not tested.

**Table 5:** Measurement invariance for sex groups. Measurement invariance of the KOOS SF-12 across sex groups.

	χ^2^	df	χ^2^_DIFF_ (df_DIFF_)	CFI	CFI_DIFF_	TLI	IFI	RMSEA
Male (n = 1787)	444.896	48	--	0.965	--	0.952	0.965	0.068
Female (n = 2132)	474.279	48	--	0.966	--	0.954	0.966	0.065
Configural Model	919.177	96	--	0.966	--	0.953	0.966	0.047
Metric Model	928.436	104	9.259 (8)	0.966	NC	0.957	0.966	0.045
Equal Variances	946.519	108	27.342 (12)	0.965	0.001	0.957	0.965	0.045
Scalar Model	1055.565	112	136.388 (16)	0.961	0.05	0.954	0.961	0.046
Equal Latent Means	1115.370	116	196.193 (20)	0.957	0.009	0.951	0.957	0.048

χ^2^_DIFF_ *p* < 0.01 or CFI_DIFF_ > 0.01; NC = no change.

**Table 6:** Measurement invariance for age groups. Measurement invariance of the KOOS SF-12 across age groups.

	χ^2^	df	χ^2^_DIFF_ (df_DIFF_)	CFI	CFI_DIFF_	TLI	IFI	RMSEA
Middle aged adults (n = 2145)	459.821	48	--	0.970	--	0.958	0.970	0.063
Older adults (n = 1073)	218.058	48	--	0.970	--	0.959	0.970	0.057
Configural	677.875	96	--	0.970	--	0.959	0.970	0.043
Metric	689.505	104	11.630 (8)	0.970	NC	0.962	0.970	0.042
Equal factor variances	696.644	108	18.769 (12)	0.970	NC	0.963	0.970	0.041
Scalar Model	775.535	112	97.660 (16)	0.966	0.004	0.960	0.966	0.043
Equal Latent Means	877.411	116	199.536 (20)	0.961	0.009	0.955	0.961	0.045

χ^2^_DIFF_ *p* < 0.01 or CFI_DIFF_ > 0.01; NC = no change.

## Data Availability

The datasets analyzed during the study are not publicly available per study protocol; however, deidentified data may be available from the corresponding author with permission from the Cedar-Sinai Office of Research Compliance and Quality Improvement, the Kerlan-Jobe Institute, and the University of Idaho upon reasonable request.
